# Characteristics and lessons learned from practice-based research networks (PBRNs) in the United States

**DOI:** 10.2147/JHL.S16441

**Published:** 2012-09-10

**Authors:** Melinda M Davis, Sara Keller, Jennifer E DeVoe, Deborah J Cohen

**Affiliations:** 1 Department of Family Medicine, Oregon Health & Science University, Portland, OR, USA; 2 Oregon Rural Practice-based Research Network, Oregon Health & Science University, Portland, OR, USA; 3 OCHIN Practice-based Research Network, Portland, OR, USA

**Keywords:** translational research, population health, participatory research, review

## Abstract

Practice-based research networks (PBRNs) are organizations that involve practicing clinicians in asking and answering clinically relevant research questions. This review explores the origins, characteristics, funding, and lessons learned through practice-based research in the United States. Primary care PBRNs emerged in the USA in the 1970s. Early studies explored the etiology of common problems encountered in primary care practices (eg, headache, miscarriage), demonstrating the gap between research conducted in controlled specialty settings and real-world practices. Over time, national initiatives and an evolving funding climate have shaped PBRN development, contributing to larger networks, a push for shared electronic health records, and the use of a broad range of research methodologies (eg, observational studies, pragmatic randomized controlled trials, continuous quality improvement, participatory methods). Today, there are over 160 active networks registered with the Agency for Healthcare Research and Quality’s PBRN Resource Center that engage primary care clinicians, pharmacists, dentists, and other health care professionals in research and quality-improvement initiatives. PBRNs provide an important laboratory for encouraging collaborative research partnerships between academicians and practices or communities to improve population health, conduct comparative effectiveness and patient-centered outcomes research, and study health policy reform. PBRNs continue to face critical challenges that include: (1) adapting to a changing landscape; (2) recruiting and retaining membership; (3) securing infrastructure support; (4) straddling two worlds (academia and community) and managing expectations; and (5) preparing for workforce transitions.

## Introduction

A practice-based research network (PBRN) is a collection of medical practices that affiliate for the purpose of conducting research focused on delivering care to the patients they serve. The Agency for Healthcare Research and Quality (AHRQ), one government agency that supports PBRN research in the United States, defines a primary care PBRN as “a group of ambulatory practices devoted principally to the primary care of patients, and affiliated in their mission to investigate questions related to community-based practice and to improve the quality of primary care.”^1^

Networks are usually formal collaborations between community-based practices and academic institutions.^1,2^ By linking questions from practicing clinicians with rigorous research methods, PBRNs can produce research findings that are relevant to clinicians and, in theory, are more easily assimilated into everyday practice. Clinicians are motivated to participate in PBRNs for many reasons: to contribute new knowledge, to reduce feelings of isolation, and to improve the quality of care provided to patients.^3–6^ Network members and PBRN structure are meant to foster a sense of commitment that transcends individual research projects.^1^

In this paper, we review the origins and development, characteristics and functions, funding, and lessons learned through research conducted in US PBRNs. We highlight how PBRNs emerged in response to the needs of practicing primary care clinicians and have adapted in parallel with changes in the health care and funding landscape. We end by exploring the role PBRNs play in building practice-based evidence, supporting research translation, and providing important infrastructure to enable change and improvement in health care delivery.

## Origins and development

**Figure 1:**
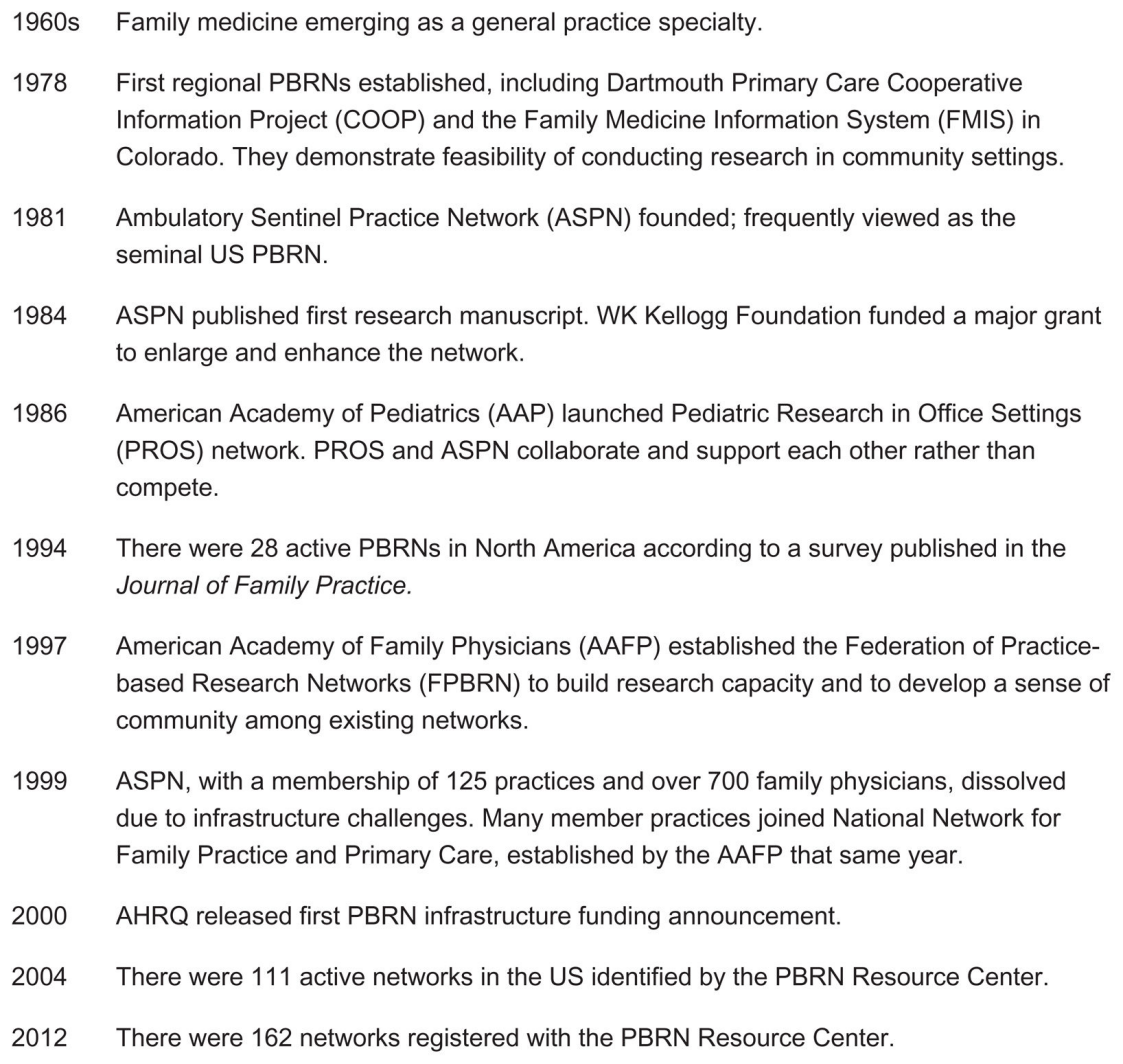
Early milestones of practice-based research network (PBRN) development in the USA.

The first regional PBRNs in the USA, the Dartmouth Primary Care Cooperative Information Project in New Hampshire and the Family Medicine Information System in Colorado, were created in 1978, slightly later than those emerging in the Netherlands, Canada, and Great Britain in the late 1960s and early 1970s.^7,8^ These US networks involved partnerships with community clinics, and they emerged at approximately the same time family medicine identified itself as a medical specialty.^9^ The Ambulatory Sentinel Practice Network (ASPN) was established in 1981; it included both US and Canadian practices. Frequently considered the seminal US PBRN, the ASPN’s research and leadership played a critical role in early PBRN development.^9,10^ A second national PBRN, Pediatric Research in Office Settings, was established in 1986 by the American Academy of Pediatrics, demonstrating the merit of these networks.^10^ Figure 1 summarizes the early milestones in PBRN development.

Although the emergence of PBRNs in the USA was slower than in European countries,^9,11^ the number of networks in the USA has grown substantially in the past few decades. According to a survey published in the *Journal of Family Practice*, there were 28 active PBRNs in North America by 1994.^12^ Early networks tended to be regional in scope and to focus on the epidemiology, natural history, and diagnosis of common problems encountered in ambulatory care.^9^ In 2002, the AHRQ supported the establishment of a PBRN Resource Center, which had over 100 primary care networks registered by 2004.^11^ Currently, the AHRQ PBRN Resource Center lists 162 registered PBRNs.^13^ The emergence of new networks has occurred in parallel with the dissolution of existing networks (eg, of the ASPN, in 1999).

## Characteristics and functions

**Figure 2:**
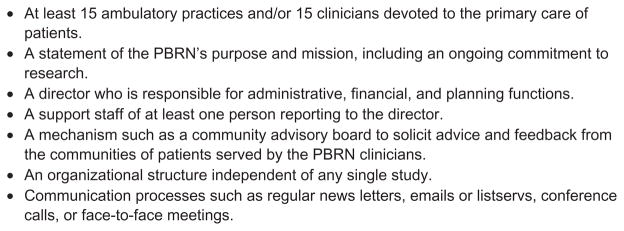
Basic characteristics of practice-based research networks (PBRNs) in the United States. **Notes:** These infrastructure elements must be in place for a PBRN to qualify for grant funding from the Agency for Healthcare Research and Quality (AHRQ) (eg, for RFA-HS-05-011 grants).

Figure 2 highlights the basic characteristics of primary care PBRNs. Data on 152 PBRNS, available as of June 2011 from the AHRQ PBRN Resource Center, indicate that these networks include over 16,900 practices staffed by 69,000 network members who provide care to approximately 53 million patients across all 50 US states. Over 90% of the registered PBRNs represent primary care networks. Forty percent of the registered networks are mixed specialty; single-specialty networks include family medicine (32%), pediatrics (12%), and internal medicine, nursing, or other practice affiliations (16%).^14^ Despite the focus on primary care, networks of dentists, pharmacists, naturopaths, and palliative medicine clinicians have also emerged in recent years.^2^ PBRNs continue to evolve in response to the needs of practicing clinicians, policy changes, and current funding opportunities.

PBRNs vary in a number of ways, including member composition (eg, single-specialty versus multispecialty), affiliation (eg, health systems, medical academies, academic institutions), and size. A recent survey of PBRNs found that 76% were affiliated with a university; most others were affiliated with a nonprofit or professional organization.^11^ Today’s PBRNs are regional (30%), state-based (28%), local (23%), and national (20%) in membership and scope.^14^ Additionally, a PBRN may have a specific mission or focus that shapes its membership requirements, such as practicing in a rural setting or in a Federally Qualified Health Center or using a specific electronic health record (EHR). Of the PBRNs registered with the AHRQ resource center, 66% use EHRs and 71% have or plan to collaborate with another PBRN.^14^ Table 1 highlights the key characteristics of five diverse PBRNs.^13,15,16^

The diversity in PBRN mission, size, and geographic area presented in Table 1 demonstrates the multiple ways networks can be designed to meet the needs of their practices, communities, and academic stakeholders. Networks may also be shaped by the expertise and interests of members and investigators in response to funding announcements, such as by developing niches in certain areas. For example, in response to shifting funding opportunities and changes in the health care landscape, some networks have embraced community-based participatory research methodology,^17,18^ focused on developing the capacity to extract or modify data from EHRs^19^ or developed expertise in conducting comparative-effectiveness research.^20^

Certain functions appear essential across PBRNs. These include supporting project development, building sustainable relationships with principal investigators and funders, recruiting and retaining voluntary clinicians and practices, managing staff and governance groups, and performing research activities (eg, developing study materials, defining human subject protocols, assisting with data management and quality control).^21^ To develop and sustain member relations, PBRNs may actively maintain a membership roster, support multiple methods of communication with key stakeholders, and host regular meetings (eg, annual member meetings).^22^ PBRNs must also keep abreast of member needs, match academic investigators with constituents who have similar topical interests, and respond to funding announcements in a timely fashion.

PBRNs create organizational structures to accomplish these functions.^21,22^ Core infrastructure frequently includes a network director (often an MD or PhD) and a coordinator who are operationally responsible for the PBRN and support the day-to-day operations of the network and research initiatives.^22^ Network leadership may also sustain an advisory board composed of representative members of the PBRN to guide network activities and inform research. To accomplish network goals and support research studies, PBRNs may hire project managers, research assistants, and statistical experts.^22^ Networks may also employ practice facilitators to assist primary care teams with quality-improvement studies, community outreach, or other shared goals.^23,24^ In some PBRNs, facilitators are regionally based, and they develop long-term relationships with member clinicians and staff that go beyond specific studies.^23–25^ Due to the affiliation of many PBRNs with academic institutions, some networks collaborate with university departments to hire core and study-specific staff for portions of their time. This can be economically beneficial for both developed and developing networks.

Variation in the structure and function of PBRNs in the USA exists because there is a dynamic interaction between these elements and the network’s mission and available resources. Green et al argued that PBRNs should establish their mission and purpose first and then design the infrastructure to support it.^22^ Early US PBRNs, as well as those today, are constantly balancing and rebalancing the infrastructure needed to maintain basic network functions and achieve their missions. The closure of the ASPN in 1999 due to inadequate infrastructure support underscores the equilibrium PBRNs must maintain.^26^

## Funding

Practicing primary care clinicians and academic faculty have contributed much in-kind effort to the development of PBRNs. However, networks also receive financial support from a number of sources, including state and federal research grants, network membership fees, and institutional and organizational affiliations. To develop as a PBRN, many networks have secured funding earmarked specifically for infrastructure development through grants or awards provided at the local or national level. Although exact figures vary with each PBRN’s research mission, estimates for annual infrastructure costs range from $69,700 for a basic network to $287,600 for a moderately complex network.^22^ Today, many US PBRNs receive funding from diverse sources, including Federal Agencies such as the AHRQ and the National Institutes of Health ([NIH] 84%), home institutions (74%), foundations (56%), professional organizations (24%), and other sources.^11,27^

**Figure 3:**
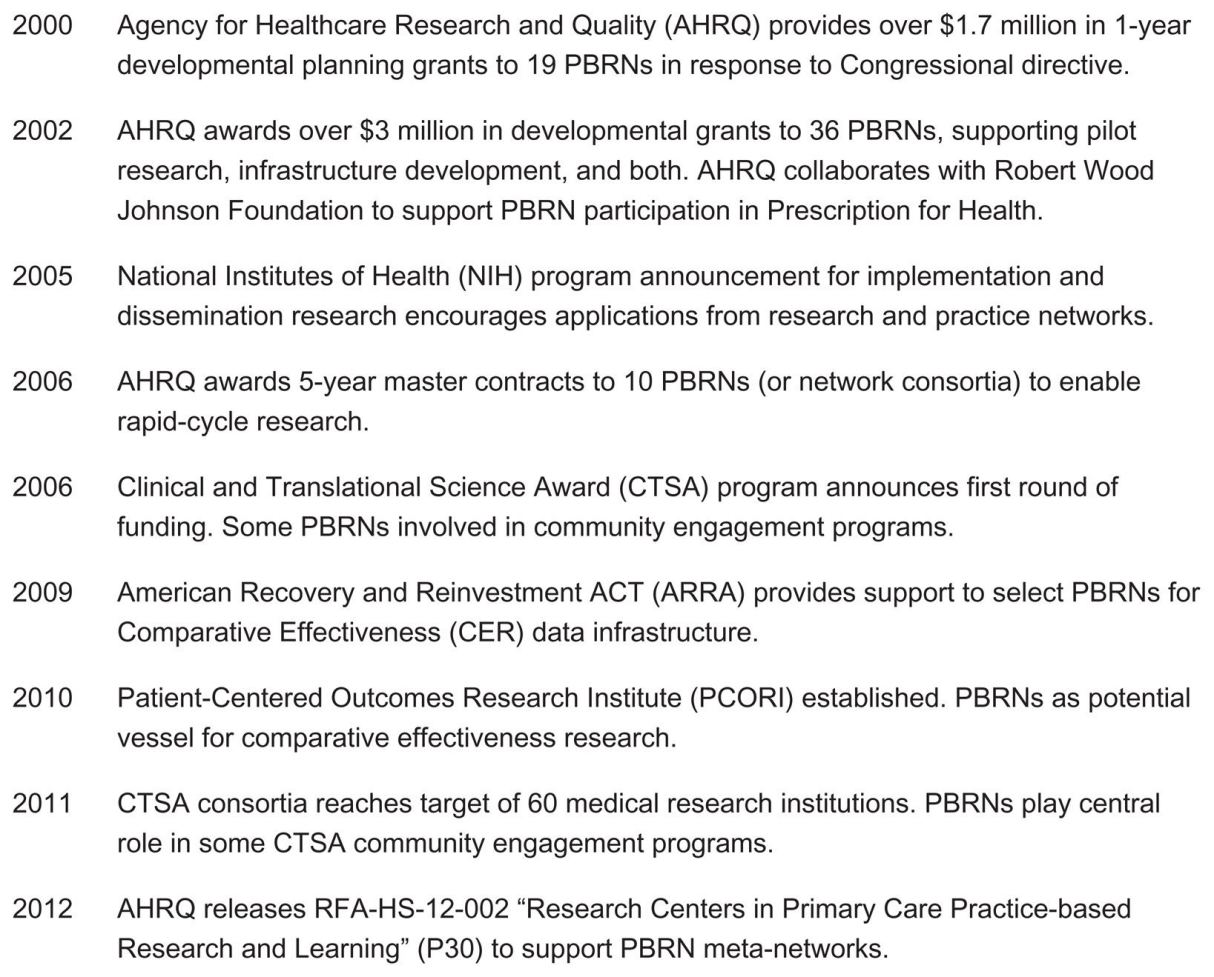
Critical opportunities for funding practice-based research network (PBRN) infrastructure and research.

Figure 3 highlights key funding opportunities that have helped support US PBRNs. PBRN growth has been encouraged through many initiatives, including at least four cycles of funding from the AHRQ, which provided developmental planning grants and capacity-building opportunities such as improving data collection by EHRs, using registries to deliver diabetes care, and enhancing the ability of clinicians and patients to participate in quality-improvement initiatives.^1^ Foundations (eg, the WK Kellogg Foundation and the Robert Wood Johnson Foundation) have also played a critical role with their support of PBRNs.^28–30^ For example, awards from the WK Kellogg Foundation allowed the ASPN to hire its first full-time staff member in 1984 and to appoint its first full-time medical director in 1985.^10^ Infrastructure and development grants, coupled with support for specific research projects, facilitated the establishment of many PBRNs and helped to sustain core operations.

The funding landscape has shaped how PBRNs frame the work they do – and it has been shaped by this research. For example, in the early 2000s, the Institute of Medicine’s Clinical Research Roundtable identified two major roadblocks to moving research into practice.^31^ The first roadblock was taking new knowledge about disease mechanisms identified through basic research and applying it to the diagnosis, treatment, and prevention of these diseases in people (eg, developing a new approach to identifying a genetic marker for breast cancer). The NIH called this “T1 research.” The second roadblock identified was translating the results of clinical studies into clinical decision making and treatment in everyday practice (eg, developing systems to ensure that all patients eligible for a colonoscopy received counseling about this test). The NIH called this “T2 research.” Many PBRNs have reframed their mission to provide community-based laboratories for T2 research. The NIH now includes dissemination and implementation research in its portfolio, which encourage applications from research and practice networks, demonstrating how PBRNs have shaped the funding landscape.^32,33^

There is also synergy between the NIH’s Clinical and Translational Science Award (CTSA) program and the newly formed Patient-Centered Outcomes Research Institute, which share many of the same priorities as PBRNs. The emergence of the CTSA program in 2006 elevated the importance of incorporating community-based research into academic health science institutions across the USA. Some PBRNs used this as an opportunity to emphasize the work they did supporting community-engaged research with both practicing clinicians and community partners. As such, CTSAs at some institutions have partnered with PBRNs to support these efforts.^34,35^ Additionally, recent funding calls from the Patient-Centered Outcomes Research Institute emphasized the importance of comparative clinical-effectiveness research to help patients and health care providers make more-informed decisions.^36,37^ PBRN infrastructure provides a critical framework for supporting research like this in real-world practice and community settings.

## PBRN research over time

PBRN research helped establish knowledge vital to the delivery of high-quality health care in ambulatory-care and community settings. PBRNs conduct research on topics that emerge from practicing clinicians (bottom-up research), and from individual investigators (top-down research).^8,11^ Some networks also use a collaborative approach by which academics and community partners (eg, practicing clinicians, patients, and organizational leadership) work together in a participatory fashion to codevelop the research agenda.^11^ We present a brief review of PBRN research over time to highlight critical contributions, describe expanding approaches and methodologies, and explore the opportunities ahead. We identified studies using a search for “practice-based research” and “practice-based research networks” in PubMed and selected a sample of articles from early, middle, and the current time periods to inform us about the lessons learned. This approach was not intended to be systematic or comprehensive, but rather to highlight patterns in the PBRN research landscape.

Early PBRN studies explored everyday clinical problems (eg, headache treatment, miscarriage treatment), and many of them engaged physicians directly in data collection using the card study methodology.^38^ Results from these initial studies were generally presented at conferences and published in the *Journal of the American Board of Family Medicine* and *Journal of Family Practice*. This research demonstrated the misalignment between evidence-based, published guidelines and the manifestation of symptoms and disease in general practice.^7,9^ For example, an observational study of usual primary care indicated that 40% of spontaneous abortions were managed completely in the office or at home. This finding raised questions about text recommendations for dilation and curettage.^39^ This and other studies highlight how the context in which you study a question (eg, in general practice or in specialty settings) shapes the answers you discover and can have a major impact on how care is rendered.^40^

These early findings made an important contribution to the evidence base in health care, and leaders in primary care used the results of these early studies to advocate for practice-based research. They did so by pointing out the limitations of randomized controlled trials that narrowly defined the study sample were conducted in controlled environments and were frequently led by researchers and specialists unfamiliar with general practice. While studies with these attributes may carry weight in the field and inform guideline-setting organizations and standards of care, they may not accurately portray the effectiveness of new treatments in the general population.

In addition to demonstrating a vital knowledge gap, early practice-based research established the feasibility of conducting research in networks of community practices.^9^ Moreover, the impact of these applied studies on clinicians and patients had the potential to be immediate and far-reaching. Green et al wrote, “The new knowledge that comes from practice-based research will not find application to only a few with fully developed or perhaps unusual disease. It will benefit virtually everyone.”^41^ Bringing practices together into a network (1) created the infrastructure to quickly get enough power or practices and people to study a problem, (2) enhanced the generalizability of the studies, and (3) increased research productivity. A single PBRN could provide the practices needed to study a range of phenomena simultaneously. Moreover, PBRNs created the infrastructure to generate practice-based evidence – evidence that is relevant to clinicians and the settings in which they practice.^11,27^

By the late 1990s, a variety of research was being done in PBRNs to look at clinical issues in obstetric,^42^ geriatric,^43^ pediatric,^44^ and family medicine settings.^45,46^ In addition to expanding research topics, PBRNs were just beginning to diversify the research methods used in studies including cross-sectional survey research,^45^ cohort studies,^42,44^ observational studies and interviews,^43^ and mixed-methods research.^46^ Much of the research coming from PBRNs at this time was published in the *Journal of Family Practice*, but findings were also beginning to gain traction in a wider range of journals, with manuscripts appearing in the *Archives of Family Medicine*, the *Western Journal of Medicine*, and the *Journal of the American Geriatrics Society*.

Just as early PBRN research studied everyday clinical problems, today’s networks explore a diverse range of phenomenon experienced in daily practice, including service delivery and health care redesign. This trend toward the expansion of PBRN research methods and research impact continues. Studies have become more complex, they involve an increasing number of participating sites, and they occur in a broader range of PBRNs, including dentistry.^47^ Studies involving multiple PBRNs,^48^ or data mined from EHRs to provide a generous sample size,^49^ are not uncommon. PBRNs can also support system-level interventions, and randomization can be done at the practice or patient level. A 2007 mixed-method study of primary care PBRN directors and administrative officers found that common research foci included prevention, diabetes, cardiovascular risk factors, and mental health.^11^ Additionally, some PBRNs play an active role in supporting health-reform initiatives and quality-improvement projects, such as assisting practices as they pursue patient-centered medical home status.^50,51^ Networks have also begun to address community-level health needs by using participatory research methods^17,52^ and conducting studies that link clinics with community-based resources to foster health behavior change.^53–55^

As network foci expanded, study participants and publication targets diversified. PBRN studies now engage various frontline health care providers, including nonphysician clinicians (nurse practitioners and physician assistants), nurses, social workers, and behavioral health specialists.^55,56^ This change highlights the increasing role of team-based care in health care settings. Additionally, articles from PBRNs now regularly appear in the *Journal of the American Board of Family Medicine*, the *Annals of Family Medicine*, and non-primary care journals, including those with a focus on medical informatics,^48^ pharmacology,^49^ health disparities,^57^ health care management,^58^ and dentistry.^47^ Top-tier journals such as the *American Journal of Preventive Medicine* have dedicated entire issues exclusively to PBRN research.^28,29^

## Lessons learned (challenges and opportunities)

PBRNs are poised to continue to play a critical role in health reform initiatives such as supporting clinic redesign,^51^ expanding partnerships between primary care and public health organizations,^59,60^ and participating in the emergence of accountable care organizations.^61^ Networks also have the opportunity to engage in comparative-effectiveness research.^20^ Networks continue to play an important role in both the study of care delivery and the application of these approaches to daily practice.^62^ We highlight five critical challenges that may inform future PBRN work.

### Adapting to a changing landscape

PBRNs have responded to the changing health care landscape by widening their membership (eg, primary care, pharmacy, ancillary staff, community partners) and embracing diverse research methodologies (eg, community-based participatory research, comparative-effectiveness research, mixed- methods research, EHR data abstraction). This expansion provides greater opportunities to partner with academic researchers, meet the needs of practicing members, and stay flexible in light of funding opportunities. Moreover, it provides the breadth and infrastructure to address critically relevant questions for practitioners, academicians, policymakers, and other stakeholders. However, network leadership may be challenged to identify and sustain a shared vision that can motivate the participation and secure the infrastructure capacity needed to respond to more diverse stakeholders.

### Recruiting and retaining membership

PBRN leadership has played a critical role in developing network membership, locating funding opportunities, and implementing research studies. Clinicians were initially drawn to PBRNs for the camaraderie and opportunity to improve care for their patients. However, changing practice structures (eg, a shift in the USA from independent clinics to system-owned sites) and growing competing demands may make it more difficult to recruit and sustain practice membership. PBRNs must learn how to negotiate their roles within larger health care systems, reframe and renew the benefits PBRNs offer individual clinicians (eg, academic scholarship), and engage nonclinician practice and systems’ administrative and executive staffs.

### Securing infrastructure support

Securing and sustaining funding to support network infrastructure will continue to be a challenge for PBRNs. Although the emergence of CTSAs provided some networks with resources to build critical, foundational relationships for research, many PBRNs still struggle to finance core infrastructure. Building a robust research capacity is difficult when networks are dependent on soft money (grant funds) for core staff support. In an increasingly competitive grant environment, networks may need to pursue nontraditional sources of funding by building partnerships with state governments, insurance companies, and health care systems. The tension between working with new partners to secure financing will need to be carefully balanced with the mission and credibility of the PBRN.

### Straddling two worlds (academia and community) and managing expectations

PBRNs play an important role in spanning the boundaries between clinical and community practice and the academic establishment. The time demands, focal concerns, and indicators of quality and achievement are different in clinical and academic environments. Practices and communities operate at a fast pace and are often focused on providing services at the individual level. Academics have accommodated a schedule of delayed gratification, where the traditional research timeline from idea to funding to project completion can last for years. Networks may struggle to balance a clinic’s and community’s needs for action with a researcher’s timeline. Developing methods to straddle these worlds for their mutual benefit may require compromise and trade-offs. For example, academics may need protected time to collaborate with clinic and community partners, an aspect of community-engaged research that is not often accounted for in traditional academic promotion calculations; practices and communities may also benefit from taking time to reflect upon research results and questions, although this is something they may have neither the patience nor the luxury to embrace.

### Preparing for workforce transitions

As with the general US population, PBRN leadership is aging. Developing transition plans so that networks can be sustained as current network directors retire will be crucial. Because many networks have a lean infrastructure, there are often no people in the ranks (eg, deputy directors) who have been mentored over time to take on leadership roles. Such transitions provide opportunities for innovation and challenge network stability.

## Conclusion

PBRNs have been identified as research laboratories essential to advancing the science of medical care. They are a venue both for describing clinical problems encountered in everyday practice and for speeding the translation of research into routine care. PBRNs have expanded from regional affiliations to national and international organizations that use multiple methods to address the needs of practicing clinicians and communities. Because PBRNs have demonstrated their effectiveness as laboratories for clinical research and knowledge translation, they have become central players in health services research. PBRNs are well poised to play important roles in implementing and exploring areas critical to health care reform, such as facilitating better integration between primary care and public health services or assisting with the development of accountable care organizations.

The sustainability and effectiveness of PBRNs have been, and may continue to be, predicated on PBRNs’ ability to negotiate their mission in light of current funding priorities and a dynamic health care environment. PBRNs must attend to five critical challenges as they move forward: (1) adapting to a changing landscape; (2) recruiting and retaining membership; (3) securing infrastructure support; (4) straddling two worlds (academia and community) and managing expectations; and (5) preparing for workforce transitions.

## Figures and Tables

**Table 1:** Network diversity: select characteristics of five US practice-based research networks (PBRNs)

PBRN name	Safety-net west PBRN	Oklahoma physicians resources/research network (OKPRN)	Mecklenburg area partnership for primary care research (MAPPR)	Pediatric research in office settings (PROS)	Practitioners engaged in applied research and learning (PEARL)
Network type	Mixed network^a^	Mixed network^a^	Family medicine	Pediatric	Dental
Mission	To improve the health of underserved populations, enhance their quality of care, and inform health policy through research using electronic health records (EHRs)	To improve health care services for clinicians in the state through research and quality-improvement initiatives in primary care and public health settings	To build a collaboration to improve health of our community by mobilizing health care professionals, community members, and researchers	To improve the health of children and enhance primary care by conducting national collaborative practice-based research	To generate ideas and conduct studies that seek research-based solutions to the problems routinely confronted by general dental practitioners
Geographic area served	Northwest region	State	University practices	National	International
Size					
Members	2455	248	300	1768	200
Clinics	157	139	97	738	N/A

**Notes:** Details are from the Agency for Healthcare Research and Quality (AHRQ) PBRN Resource Center^13,15^ and the PEARL website.^16^

a Combination of family medicine, internal medicine, pediatrics, nursing, or other specialties.
